# Results of a one year survey of output for linear accelerators using IMRT and non‐IMRT techniques

**DOI:** 10.1120/jacmp.v5i1.1960

**Published:** 2004-05-25

**Authors:** James G. Mechalakos, Jean St. Germain, Chandra M. Burman

**Affiliations:** ^1^ Department of Medical Physics Memorial Sloan‐Kettering Cancer Center 1275 York Avenue New York 10021

**Keywords:** radiation therapy, workload, IMRT, linear accelerator, shielding

## Abstract

This paper presents the results of a one year survey of treated fields for 3 treatment machines at our New Jersey regional center. One machine, predominantly, treated IMRT prostate patients using a sliding window technique. The others were not equipped to deliver IMRT. Information obtained for each treated field included patient number, modality, monitor units delivered, gantry angle and time. Data was obtained directly from our record and verify system, and analyzed using a spreadsheet. We studied workload (MU/wk), patient load and average MU per patient as a function of time as well as angular distributions and number of treatment fractions per patient. We also calculated the fraction of time the beam was on during treatments.

By the end of the survey year, the workload of the IMRT machine reached 100,000 MU/wk, approximately, and that of the non‐IMRT machines, around 40−45000 MU/wk. This was largely due to the higher number of monitor units for IMRT plans. Patient loads were not significantly different for the 3 machines. Duty cycle was 14% and 16% for the non‐IMRT machines, and 27% for the IMRT machine.

The difference in workload for IMRT treatments relative to non‐IMRT treatments confirms an earlier study performed at our institution using a much smaller data sample. One needs to consider the increase in leakage associated with this higher workload when designing shielding for an IMRT room.

PACS numbers: 87.52.Df, 87.53.St

## I. INTRODUCTION

IMRT, or Intensity Modulated Radiation Therapy, has proved to be an effective treatment technique for a number of sites, and continues to grow in popularity and availability. A number of different techniques exist for implementing IMRT, one of the most common being the sliding window technique in which the desired fluence profile of a particular beam is delivered using moving leaves of a multi‐leaf collimator (MLC). This technique creates a moving aperture which delivers the prescription dose over a longer period of time as compared to a conventional open field. Another technique, called the “step‐and‐shoot” method, delivers the dose using a set of static fields, the sum of which yields the desired intensity profile.

It is proved (as shown in a previous report from our institution) that a machine treating, primarily, with sliding window IMRT has a workload which is, approximately, a factor of 2 higher than non‐IMRT machines.^1^ Based on these results, we concluded that the workload recommendations of the NCRP were sufficiently conservative.^2^ ^3^ ^4^ A point to note is the NCRP recommendation of 100,000 MU's per week for a busy institution treating below 10 MV assumed a patient load of 50. Therefore, while the workload estimate of the NCRP may have been sufficiently conservative, the energy dependence and assumed patient load is dated.

This increase in monitor units does not appreciably affect the amount of radiation reaching the primary barrier on a per‐plan basis, since the prescription dose, and consequently, the amount of radiation reaching the primary barrier, stays the same. If, however, more patients are treated per day, due to less time between fields, the total weekly dose to the primary barrier will increase. Therefore, it would be instructive to examine not only the increase in monitor units associated with an IMRT plan, but also the increase in patient load and duty cycle, which we shall define as the fraction of time that a machine is “on” during treatment hours. The dose reaching the secondary barrier will be more significantly affected, as this dose is more dependent on the beam‐on time and not on the dose to the target. Leakage radiation exists whether photons are being used or electrons. Therefore, it would be most instructive to take them both into account.

In our last report, we examined a small sample of the overall number of patient charts. In this study, we wish to obtain a clearer picture of the contribution of IMRT to machine workload, and to look at patient loads and duty cycles. Therefore, we extracted one year's worth of data from our record and verify system, including treatment times and number of monitor units for every field treated that year. Thereby, we were assured a very accurate representation of monitor units delivered as a function of time. Three machines were surveyed for this report–located at MSKCC at St. Clare's, our New Jersey regional centers in Dover and Denville (two clinics within a short distance from each other, sharing the same staff). A total of 63,698 fields were treated, using the 3 treatment machines. The overall workload of the machines included these treatments plus films. Workload associated with QA procedures was not included in the calculation as it was performed during off‐hours, and therefore, not recorded in the record and verify database.

## II. MATERIALS AND METHODS

Memorial Sloan‐Kettering Cancer Center at St. Clare's Hospital is a regional clinic of Memorial Sloan‐Kettering Cancer Center [MSKCC]. Being part of the MSKCC network, the same treatment protocols are observed as at the main campus in New York City with regard to conventional and IMRT treatments. The clinic is equipped with 3 treatment machines:

- A multi‐energy Siemens Mevatron KD, which has no MLC, and is equipped with a beam stop that is used for fields in which the gantry angle is between 50 and 310 degrees;
- A single energy Varian 600C with a 52 leaf MLC which was not equipped for IMRT treatments at the time of this survey; and
- A Varian 2100C, equipped with an 80‐leaf dynamic multileaf collimator with leaf width of 1 cm capable of delivering IMRT treatments using the sliding window technique.

A summary of machine parameters is given in Table I.

**Table I:** Summary of treatment machine parameters

Linac	Energy (MV/MeV)	IMRT	Predominant technique or site treated	No. fields treated	% photon treatments
Siemens Mevatron KD	Photons: 6, 15 Electrons: 6, 9, 12, 15, 18, 21	No	2D, standard angles	15,846	84
Varian 600C	Photons: 6 MV	No	Breast	15,491	100
Varian 2100C	Photons: 6, 18 Electrons: 6, 9, 12, 16, 20	Yes	IMRT prostate	32,631	97

The Siemens machine treated a total of 15,846 fields for the year, 84% of which used photons (a treatment, as opposed to a film, was counted as any field to which over 10 monitor units were delivered). The 600C unit treated a total of 15,491 fields for the year and used only photons (breast patients on the 600C received their electron boosts on the Siemens unit, if prescribed). The 2100C unit treated a total of 32,631 fields, 97% of which used photons.

The only anatomical site treated using IMRT during this survey was the prostate. At MSKCC, a standard set of beams is used for typical IMRT prostate treatments, at angles of 0, 75, 135, 225 and 285 degrees (IEC scale). Angles may be shifted slightly for special cases. The prescribed dose is, typically, 8100 cGy or 7020 cGy for post‐operative treatments of the prostate bed, always in 180 cGy fractions. Patients are typically treated in the prone position.^5^

During the time period over which data was gathered for this report, all 3 machines were using a record and verify system developed at MSKCC, called CASPER (Computer Assisted System for Patient data Entry and Retrieval), which not only scheduled and monitored treatments, but also stored relevant treatment parameters. Therefore, it was possible to extract machine parameters for all past treatments associated with a particular machine. In cases where patients had to be treated off line, due to equipment or network problems, therapists were required to manually enter the treatment parameters into CASPER; as a result, the CASPER database stored information on all treated fields. CASPER data was stored on Alpha workstations (Digital Corp.), and had to be first transferred to PC, using FTP. Once it reached the PC, it was imported in its raw form into a Microsoft Excel spreadsheet and a macro was used to format it. Beam‐on time from quality assurance procedures was not stored in the CASPER database.

Treatment data was extracted from the CASPER database for all 3 treatment machines, for a period of one year, from August 2000 to August 2001. This period was interesting, because the 2100C had recently begun treating using IMRT and was, therefore, increasing its workload as more patients were accrued. For each field treated, the data included the date and time of treatment, patient name and ID, number of monitor units (defined at our institution as 1 MU=1 cGy delivered to water at a depth of $d_{\max}$ for a 10 cm×10 cm field), treatment mode (photon or electron), energy and gantry angle. Since we were more concerned with leakage contributions to the secondary barriers than with primary dose contribution to the primary barriers, data was included for all treatments. Once the data was in the spreadsheet and formatted, a number of macros were written to analyze the data.

Rather than calculating true use factors associated with the geometry of the room, we created a histogram of the total output for each gantry angle using 1 degree bins. All monitor units for a particular treatment were added to the central axis angular bin, and not spread out over the angular width of the field.

In order to calculate workload in terms of monitor units delivered, we determined the total output per week of treatment. The workloads for the 3 machines were then inter‐compared. We also determined the number of patients on treatment per day for each machine as well as the average number of monitor units per patient per day, to deconvolve the patient load from the output. Finally, on the IMRT machine, we determined the daily fraction of patients treated with IMRT.

The average duty cycle of each machine, which we defined as the average fraction of time the beam was on during patient treatments, was calculated as follows. For each field *i* treated, the duty cycle $D_{i}$ was defined as

(Equation 1) $$D_{i}\;=\;\begin{array}{l} \frac{\text{MU}\;\;\;\;\;\;\;\;\;\;\;\;\;\;\;\;\;\;\;\;\;\;\;\;\;\;\;\;\;\;\;\;\;\;\;\;}{(t_{i+1}-t_{i})\times DR} \end{array}$$

where

1. MU=the number of monitor units delivered
2. $t_{i}=\text{the time that the field was treated}$
3. $t_{i+1}=\text{the time that the field was treated}$
4. DR=the dose rate used for the treatment.

The Varian 2100C ran at a typical dose rate of 240 MU/min, and the Varian 600C ran at a typical dose rate of 250 MU/min; while the Siemens unit ran at a typical dose rate of 240 MU/min for 6 MV photons, and 300 MU/min for 15 MV photons and all electron energies. The last treatment of the day was not included in the calculation since the next treatment was on the following day, giving a duty cycle near zero, according to the definition. Given that an average of 82, 80 and 171 fields/day were treated on the Siemens, Varian 600C and Varian 2100C, respectively, the effect of not including the last field of the day in the duty cycle calculation was negligible. Also, some treatments were entered manually when the record and verify system was down. These treatments had a duty cycle larger than one, since the time recorded is the time of entry and not the time of actual treatment. These were not included in the calculations and comprised less than 2% of all the entries.

The average treatment duty cycle for the machine was an average of the duty cycles for all treatments:

(Equation 2) $$〈D〉=\frac{\;\;\;\;\;\;\;\sum_{i=1,}^{N}\underset{N}{D_{i}}}{N\;\;\;\;\;\;\;\;\;\;\;\;\;\;\;\;\;}$$

where *N* is the total number of treatments for the year. Films were not included.

## III. RESULTS

### Output vs Gantry Angle

**Figure 1:**
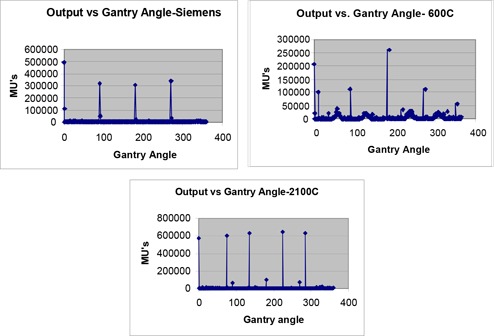
Output vs gantry angle (IEC scale)

Histograms of output vs gantry angle for the 3 machines are shown in Fig. 1. This figure can yield information about predominant types of treatment with unique gantry angles, especially for IMRT treatments. The output vs gantry angle histogram for the Siemens machine showed marked peaks at the 4 “standard” angles, reflecting that this machine treats, predominantly, standard 4 field cases, viz. AP/PA treatments and two lateral fields. Besides the standard angles treated by the 600C, the four smaller peaks at approximately 60, 140, 230 and 310 degrees represent tangential breast fields. The peaks at 10 degrees and 350 degrees represent supraclavicular fields treated with certain breast plans, tilted 10 degrees from the vertical in either direction so as to avoid the spinal cord.

The output vs gantry angle plot for the 2100C showed 5 distinct peaks at 0, 75, 135, 225 and 325 degrees. These are the 5 standard angles used to treat IMRT prostate patients. The dominance of these angles reflects the strong contribution made by IMRT to the workload of this machine.

### Weekly output

Weekly output is dependent on a number of factors, patients per day and monitor units per treatment being most important.

**Figure 2:**
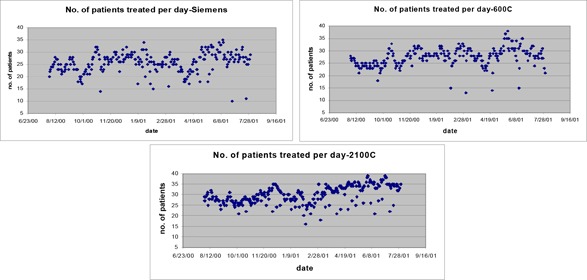
Number of patients treated per day. Days with less than 5 patients not shown.

The number of patients per day increased slightly over the year, but was comparable, as shown in Fig. 2. The patient load on the Siemens machine increased from 20 to 30 patients per day, approximately. The load on the Varian 600C increased from 25 to 30 patients per day, approximately, and the Varian 2100C load increased from 25 to 35 patients per day, approximately. The 2100C had the highest patient load, although not appreciably higher.

**Figure 3:**
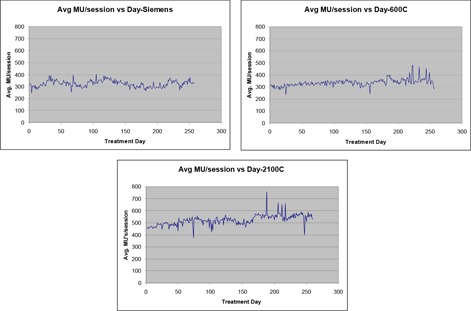
Average number of monitor units per treatment session per day.

The average number of MU's per treatment session as a function of time is shown in Fig. 3. The average number of monitor units per session varied between 300 and 400 MU's, approximately, for the non‐IMRT machines. The average number of monitor units per session increased throughout the year from 450 to 550, approximately, on the 2100C.

**Figure 4:**
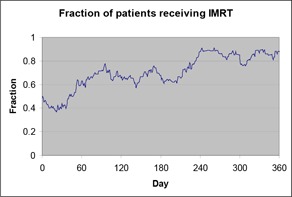
Fraction of patients receiving IMRT treatments per day

The increase in the average number of monitor units per day on the 2100C can be associated with the increase in the fraction of patients receiving IMRT on that machine, as shown in Fig. 4, which shows an increase from 50% to 90% of patients, approximately. (IMRT treatments had begun in early 2000.)

**Figure 5:**
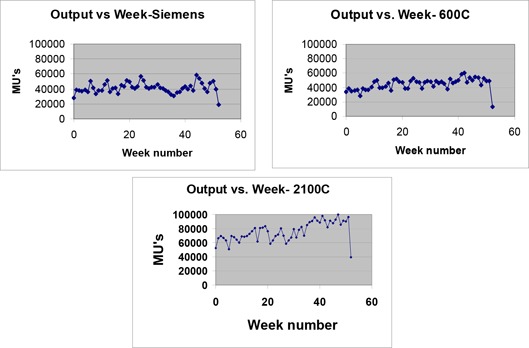
Treatment machine output vs. week. The last datum represents a partial week.

The clinically significant result of the above factors can be seen in the weekly output for the 3 machines given in Fig. 5. The Siemens and the Varian 600C showed weekly outputs averaging 40,000 MU/wk and 45,000 MU/wk, respectively; however, the Varian 2100C ranged from 60,000 MU/wk at the beginning of the year to almost 100,000 MU/wk by the end of the year.

### Duty cycle

The average treatment duty cycle of the Varian 2100C was the highest, at 27%. The Siemens machine had the lowest average duty cycle, 14%, while the Varian 600C had an average duty cycle of 16%. In our case, therefore, the predominant IMRT machine had an average duty cycle that was 11%‐12% higher than non‐IMRT machines.

## IV. DISCUSSION

This report confirms our previous results, namely, that IMRT increases the machine workload to a significant extent. Therefore, the use of IMRT must be considered in shielding calculations. In this study, where the only site being treated with IMRT was the prostate, the workload of the IMRT machine at the end of the year was approximately a factor of 2 higher than that of the non‐IMRT machines. The higher workload associated with IMRT techniques can be due to both a higher number of monitor units per plan and a higher number of patients treated on the machine. Indeed, the fact that a limited number of machines may be available to treat with IMRT techniques in a particular department, combined with the demand for IMRT treatments, may result in a heavier patient load for IMRT machines in comparison to non‐IMRT machines. Our study showed a slightly higher patient load on the IMRT machine vs the other two machines; however, the higher number of monitor units per plan was primarily responsible for the higher workload.

The increase in workload associated with the arrival of IMRT techniques must, therefore, be predicted not only on the basis of the type of plans expected, but the availability of IMRT as a function of demand and the planned reallocation of certain types of treatments to other machines once IMRT techniques are implemented.

Our study showed a difference of over 10% in average treatment duty cycle (defined as the average percentage of time the machine is on during a treatment session) on the machine equipped for IMRT as compared to the other 2 machines. This is partly due to the fact that therapists do not have to travel in and out of the treatment room with most IMRT patients to change wedges or blocks. The calculation is only an estimate based on the information available. Filming (any field to which 10 MU's or less was given) was not included; however, gaps between successive patients, though rare, may have had a small effect. Also, manual entry of monitor units resulted in entries for which large numbers of MU's per field were given in very short times, resulting in duty cycles greater than 1. These were also excluded.

The dose to the secondary barriers, which is proportional to the workload, will increase, as will the neutron leakage contribution for higher energy treatments. Since treatment consoles are typically behind a secondary barrier, one could examine the personnel monitors of therapist personnel to assess what effect, if any, exists. Given the fact that therapists work in shifts of 8 hours per day, or approximately 7 hours in front of the console, a single therapist would not be expected to receive more than 70% of the leakage radiation in that area, assuming a 10 hour treatment day.

We had concluded in our previous work that the NCRP estimated workload of 100,000 rad/wk at isocenter for a busy institution was adequate. Since the results of this study are comparable to the previous study, we continue to believe that an estimate of 100,000 MU/wk for a busy department is reasonable for sliding window IMRT, such as ours. It is important for each institution to evaluate the potential workload increase based on the IMRT technique they wish to use, combined with the expected patient loads. Other institutions have reported increases in the number of monitor units per plan associated with IMRT techniques, using other methods. For an overview of some of these results, see the IMRT Collaborative Working Group report.^6^

As described in this report, data from the on‐site record and verify system can be extracted and analyzed with a spreadsheet program to properly assess the workloads of each machine in an expedient fashion. A department beginning an IMRT program can assess changes in workload, before and after implementation of IMRT, on the upgraded machine as well as other machines in the department.

## ACKNOWLEDGMENTS

The authors thank Michael Sullivan and Yim‐Ling Dinstl of Memorial Sloan Kettering Cancer Center, Department of Radiation Oncology, for extraction of record and verify data used in this analysis.
